# Microbiota alteration of Chinese young male adults with high-status negative cognitive processing bias

**DOI:** 10.3389/fmicb.2023.989162

**Published:** 2023-03-01

**Authors:** Hui-Min Xu, Shen-Wei Xie, Tian-Yao Liu, Xia Zhou, Zheng-Zhi Feng, Xie He

**Affiliations:** ^1^Department of Medical Psychology, School of Psychology, Army Medical University, Chongqing, China; ^2^Taiyuan Satellite Launch Center, Taiyuan, China; ^3^The People’s Liberation Army (PLA) 953 Hospital, Army Medical University, Rìkazé, China; ^4^Daping Hospital, Army Medical University, Chongqing, China

**Keywords:** negative cognitive processing bias (NCPB), cognition, microbiota, depressive symptoms, *Faecalibaculum*, Chinese young male adults

## Abstract

**Introduction:**

Evidence suggests that negative cognitive processing bias (NCPB) is a significant risk factor for depression. The microbiota–gut–brain axis has been proven to be a contributing factor to cognitive health and disease. However, the connection between microbiota and NCPB remains unknown. This study mainly sought to explore the key microbiota involved in NCPB and the possible pathways through which NCPB affects depressive symptoms.

**Methods:**

Data in our studies were collected from 735 Chinese young adults through a cross-sectional survey. Fecal samples were collected from 35 young adults with different levels of NCPB (18 individuals were recruited as the high-status NCPB group, and another 17 individuals were matched as the low-status NCPB group) and 60 with different degrees of depressive symptoms (27 individuals were recruited into the depressive symptom group, as D group, and 33 individuals were matched into the control group, as C group) and analyzed by the 16S ribosomal RNA sequencing technique.

**Results:**

As a result, the level of NCPB correlated with the degree of depressive symptoms as well as anxiety symptoms and sleep quality (*p* < 0.01). The β-diversity of microbiota in young adults was proven to be significantly different between the high-status NCPB and the low-status NCPB groups. There were several significantly increased bacteria taxa, including Dorea, *Christensenellaceae*, *Christe -senellaceae_R_7_group*, *Ruminococcaceae_NK4A214_group*, *Eggerthellaceae*, *Family-XIII*, *Family_XIII_AD3011_group*, *Faecalibaculum*, and *Oscillibacter*. They were mainly involved in pathways including short-chain fatty acid (SCFA) metabolism. Among these variable bacteria taxa, *Faecalibaculum* was found associated with both NCPB and depressive symptoms. Furthermore, five pathways turned out to be significantly altered in both the high-status NCPB group and the depressive symptom group, including butanoate metabolism, glyoxylate and dicarboxylate metabolism, propanoate metabolism, phenylalanine, tyrosine, and tryptophan biosynthesis, valine, leucine, and isoleucine degradation. These pathways were related to SCFA metabolism.

**Discussion:**

Fecal microbiota is altered in Chinese young male adults with high status NCPB and may be involved in the biochemical progress that influences depressive symptoms.

## Introduction

From the cognitive perspective, negative cognitive processing bias (NCPB), which was characterized as negative bias in attention, explanation, memory, and rumination being accompanied by poor sleep quality, has been reported to be the core feature of depression (Disner et al., 2011; Rozenman et al., 2014; Gobin et al., 2015). The information processing involving sensory, perception, attention, memory, learning, and so on constitutes cognition, which has been proven to be essential for maintaining physical and mental health (Kwak et al., 2016; Bayne et al., 2019; Liu et al., 2020; Ling et al., 2021). For the last decade, the cognitive(Aatsinki et al., 2022) function has been linked with microbiota composition. Initially, germ-free (GF) mice were found to exhibit anxiolytic basal behavior utilizing the elevated plus maze (EPM) compared to the specific pathogen-free (SPF) mice (Neufeld et al., 2011). Furthermore, GF mice were proven to be exhibiting changes in learning, memory recognition, and emotional behavior resulting from the absence of microbiota (Connell et al., 2022). The studies on the fecal microbiota of 8-month-old infants described the association between early microbiota and later fear bias. It observed a lower abundance of *Bifidobacterium* and a higher abundance of *Clostridium* with an increased “fear bias” of infants. Chronic antibiotic depletion of microbiota populations alters cognition-related metabolism and the expression of key cognitive signaling molecules, leading to long-lasting effects on cognition (Fröhlich et al., 2016; Connell et al., 2022). The administering probiotics, such as *Bifidobacterium longum* 1714, modulate the behavior or cognition in both rodents and humans (Savignac et al., 2015; Akbari et al., 2016; Connell et al., 2022). Novel perspectives suggest that the dynamic bidirectional communication systems of the microbiota–gut–brain axis may be a contributing factor to cognitive health and diseases. Still, the exact mechanisms, especially on NCPB, remain unknown (Connell et al., 2022).

Traditionally, neurobiological mechanism holds the view that a loss of neural plasticity explains the occurrence of depression (Duman et al., 1999; Li and Wang, 2021). At present, abnormality of the microbiota–gut–brain axis is verified as an important risk factor for depressive symptoms (Aizawa et al., 2016; Caspani et al., 2019; Chung et al., 2019; Berg et al., 2020). The germ-free mice were found to decrease the immobility time in the forced swimming test more than healthy control mice. Furthermore, fecal microbiota transplantation of GF mice derived from patients with major depressive disorder (MDD) leads to aggravating depressive-like behaviors compared with the colonization of the “health” fecal microbiota from control individuals (Zheng et al., 2016). According to Beck’s cognitive theory of depression, individuals suffering from stressful life events might automatically activate negative cognitive schemas, which lead to a negative tendency of cognition (Beck, 1967). The unpredictable chronic mild stress (UCMS) model mouse displays depressive-like behaviors. Fecal microbiota transplantation could have transferred the depression phenotype from UCMS donor mice to naive recipient mice (Chevalier et al., 2020). The studies have revealed that the adverse effects of UCMS-transferred microbiota were alleviated by complementation with a strain of the *Lactobacilli genus* in the mice (Chevalier et al., 2020). Yet the microbiota–brain–gut axis of NCPB has been largely neglected.

The purpose of our study was to identify the adverse effect of NCPB on an individual’s depressive symptoms, explore the role of microbiota in NCPB, and discuss the possible underlying mechanisms by which NCPB affects depression. We evaluated the changes in fecal microbial community structure and composition in subjects with high-status NCPB or depressive symptoms, analyzed the correlation of the variation tendency involved in NCPB with the degree of depressive symptoms, and hypothesized that (a) NCPB positively correlated with depressive symptoms, anxiety symptoms, and poor sleep quality in young adults; (b) the changes in microbiota could be observed in individuals with high-status NCPB, as well as depressive symptoms compared to that in controls; and (c) specific taxa and functional pathways may be found to potentially mediate the affection of NCPB on depressive symptoms.

## Materials and methods

### Study design and participants

#### Self-report measures

The self-designed socio-demographic information questionnaire was used to collect personal information including participants’ age, gender, ethnicity, only-child status, family types, and contact ways. Moreover, based on Beck’s NCPB theory of depression, the negative cognitive processing bias questionnaire (NCPBQ) is designed to assess the degree of an individual’s NCPB (especially for the Chinese population). In our previous studies, NCPBQ has good reliability (Cronbach’s alpha coefficient = 0.89) and validity (all factor loads are over 0.30) in the Chinese population (Yan et al., 2017). It showed good reliability in this study with Cronbach’s alpha coefficient being 0.92 (Jiang et al., 2017; Huang et al., 2021; Li et al., 2021). It is a 4-point scale on four factors: negative attention bias (i.e., sustained attention on negative information), negative memory bias (i.e., tendency to remember or recall negative life events), negative rumination bias (i.e., rumination on personal negative emotions), and negative explanation bias (i.e., tendency to make a negative explanation on events). The total scores of NCPBQ range from 16 to 64, with the higher scores indicating the higher status of NCPB. In the present study, to recruit participants with a high or low level of NCPB, we transformed the linear slope from a continuous variable into three categories on a common way of grouping (bottom 27%, top 27%, and others) (Notebaert et al., 2020; Ge et al., 2022).

Self-Rating Depression Scale (SDS) is a 4-point Likert-type and self-reported scale used to assess the severity of depressive symptoms. A total raw SDS score was obtained by summing the ratings of the 20 items, which were divided by 80 to create a depression severity index. A depression severity index greater than 0.5 was considered to indicate depressive symptoms. With high internal consistency, high validity in differentiating between depressed and non-depressed subjects, and international propagation, SDS has been a worldwide inventory among the most used self-rating scales for measuring depressive symptoms (Zung, 1965).

Anxiety symptoms were assessed by self-report with the Chinese version of the Self-rating Anxiety Scale (SAS), which has been validated in the Chinese population in several studies (Zung, 1971; Gong and Chan, 2018; Wu et al., 2019). Cronbach’s alpha coefficient measured in the current study was 0.79.

Pittsburgh Sleep Quality Index (PSQI) is a measurement tool used for assessing sleep quality, which has been widely used in clinical and healthy populations all over the world. The higher scores indicate poorer sleep quality. A Chinese version of the PSQI has been validated with adequate reliability (Buysse et al., 1989; Guo et al., 2016).

### Study design and participants

**FIGURE 1:**
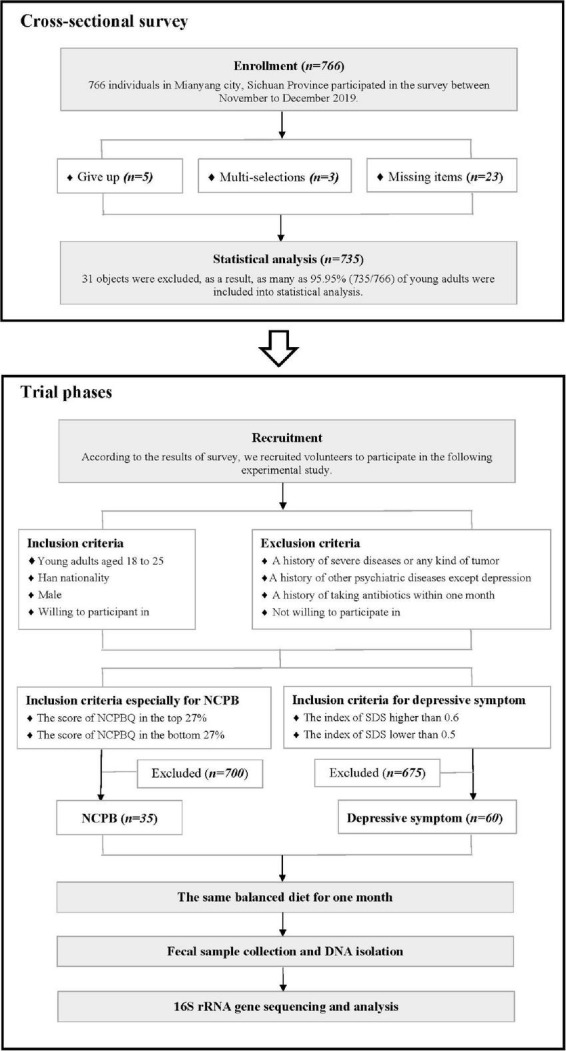
Flow diagram of the study. HS group, the high-status NCPB group; LS group, the low-status NCPB group; D group, the depression symptom group; C group, the control group.

The study design was approved by the Human Research Ethics Committee of the Army Medical University (the number was: IEC-C-[B013]-02-J.02). A cross-sectional survey was conducted in Mianyang city, Sichuan province, between November and December 2019, which covered 766 individuals. According to the results of the survey, 18 young adults with high-status NCPB and 17 with low-status NCPB (HS represented the high-status NCPB and LS represented the low-status NCPB) were recruited in the following experimental study. Meanwhile, 27 people with severe depressive symptoms and 33 people as the control were also recruited. In the trial phase, fecal samples were collected to analyze the structure of fecal microbiota after subjects were given 1 month of the same balanced diet. The detailed design is shown in Figure 1. Written informed consent was obtained from all participants.

In the cross-sectional survey, eligible subjects are defined as follows: (1) young adults aged 18–25 years old and (2) young adults who were willing to take part in the survey. All subjects consumed the same balanced diet according to Chinese Recommended Dietary Allowance (Ge, 2011; Yang et al., 2018) for 1 month (Sanz, 2010; Jing et al., 2021) to avoid the influence of diet and forbidden snacks. A total of 766 individuals were approached for participation, and 31 of them were excluded from our analysis. Finally, as many as 95.95% (735/766) of young adults were included in the statistical analysis.

In the experimental study section, 18 individuals were recruited as the HS group if their scores of NCPBQ were in the top 27%. Similarly, another 17 individuals were matched as the LS group with scores of NCPB in the bottom 27% as in previous studies (Notebaert et al., 2020; Ge et al., 2022). Moreover, 27 individuals with an SDS index (see later) higher than 0.6 were recruited into the depressive symptom group (D group), and 33 individuals lower than 0.5 were matched as the control group (C group) (Zung, 1965). Considering that a variety of factors might influence microbiota composition, we excluded subjects in the previous 1 month if any of the following criteria were met, including (1) a history of severe cardiac, pulmonary, hepatic, renal, intestinal diseases, or any kind of tumor; (2) antibiotic, probiotic, prebiotic and synbiotic application, as well as active bacterial, fungal or viral infections, gastrointestinal surgery; (3) a history of other psychiatric diseases except depression (e.g., schizophrenia); and (4) they were not willing to participate in the experiment. All the subjects needed to meet the following conditions: (1) young adults aged 18–25; (2) Han nationality; (3) male; and (4) they were willing to take part in the experiment.

### Cross-sectional survey

The cross-sectional survey was carried out after a clear illumination. The participants were asked to complete the questionnaires designed for collecting personal information and estimating the degree of NCPB, depressive symptoms, anxiety symptoms, and sleep quality.

### Fecal sample collection and DNA isolation

According to the results of the cross-sectional survey, 18 young adults with high-status NCPB and 17 age- and gender-matched with low status were recruited. Moreover, 27 young adults with high depressive symptoms were selected into the depressive symptom group, and 33 age- and gender-matched young adults with low depressive symptoms were also recruited. The fecal samples collected from the recruited participants in the morning were numbered and stored at −80°C before analyses. According to the instruction, the standard Power Soil DNA Isolation Kit was used to extract DNA (QIAGEN, Germany).

### 16S rRNA gene sequencing and analysis

The V3–V4 variable region of the bacteria’s 16S ribosomal RNA (rRNA) gene was amplified by PCR amplification technology with barcode-indexed primers, including 338F (5′-ACTC-CTACGGGAGGCAGCA-3′) and 806R (5′-GGACTACHVGGGTWTCTAAT-3′). The PCR amplification system and reactions were consulted in the previous studies (Zhuang et al., 2018). In the experimental section, raw reads were filtered and quality-controlled to remove chimeric sequence reads. All remaining sequence reads were assigned to operational taxonomic units (OTUs) with a 97% threshold of pairwise identity using the UCLUST comparison tool of the quantitative insights into microbial ecology (QIIME) pipeline (version 1.8.0^1^) and then taxonomically classified with the RDP reference database. α-Diversity including the ACE index, Chao index, Simpson index, and Shannon diversity index was calculated for each sample or group. β-Diversity was also evaluated by principle coordinate analysis (PCoA) of weight and unweight UniFrac distances and Bray–Curtis dissimilarity as previously described (Chung et al., 2019). Furthermore, the taxonomic distributions of OTUs were performed and graphics were constructed based on the relative abundance of microbiota in each taxon in the samples and groups. The linear discriminant analysis (LDA) effect size (LEfSe) method was used to identify significant OTUs differentially. Moreover, Mann–Whitney U-test was conducted to assess differences in socio-demographic characteristics, species diversity indexes, and the relative abundance of microbiota among the various taxonomic levels using SPSS 22.0 (IBM, Armonk, NY, USA). Finally, the KEGG database^2^ was used in the signal pathway analysis to annotate pathways. We analyzed functional pathways by STAMP (version 2.1.3) (Parks et al., 2014) to explore the potential functional properties of the identified microbiota. All tests mentioned earlier were two-tailed, with a *p*-value of < 0.05 considered statistical significance.

### Statistical analysis

In the analysis of cross-sectional survey results, reliability and internal consistency were estimated using Cronbach’s alpha coefficient. Descriptive statistics, *t*-test, one-way ANOVA or Chi-square test, Pearson’s correlation analysis, and logistic regression analysis were performed with SPSS 22.0 (IBM, Armonk, NY, USA).

## Results

### Results of cross-sectional survey

A total of 735 young adults were finally enrolled in the statistical analysis. Those subjects were all male adults, ranging from 18 to 26 years old (23.76 ± 3.67). A majority of them were Han ethnicity, who showed less depressive or anxiety symptoms and better sleep quality (*p < 0.05). Detailed socio-demographic variables are shown in Supplementary Table 1.

Negative cognitive processing bias is positively correlated with depressive symptoms and anxiety symptoms, as shown in Table 1. Correlations among study variables showed that NCPB positively correlated with depressive symptoms, anxiety symptoms, and sleep quality in young adults. It was also found in the relationship between depressive symptoms, anxiety symptoms, and sleep quality. The results of regression analyses showed that NCPB. could positively predict the degree of depressive symptoms and anxiety symptoms and negatively predict the quality of sleep, covering the proportion of total variance of 16.7, 31.8, and 20.5%, respectively. Detailed information is shown in Table 2.

**TABLE 1:** Correlation among study variables (*r*).

Variable	NCPB	Depressive symptoms	Anxious symptoms	Sleep quality
NCPB	–			
Depressive symptoms	0.41**	–		
Anxious symptoms	0.56**	0.70**	–	
Sleep quality	0.45**	0.41**	0.52**	–

**p < 0.01.

**TABLE 2:** Regression analysis of NCPB on depressive symptoms, anxiety symptoms, and sleep quality.

Variable	*B*	*SE*	β	*t*	*p*
Depressive symptoms	0.32	0.03	0.41	12.13	<0.01
Anxious symptoms	0.35	0.02	0.56	18.50	<0.01
Sleep quality	0.13	0.01	0.45	13.69	<0.01

### Comparison of the microbiota in the high-status and low-status NCPB groups

As shown in Supplementary Table 2, no significant difference was found in gender, ethnicity, age, only-child status, and the body mass index (BMI) between the high-status and low-status NCPB. We have already verified the sample size of flora analysis and the data collection volume is sufficient before the comparision of the microbiota in different groups, as shown in Supplementary Figure 1.

**FIGURE 2:**
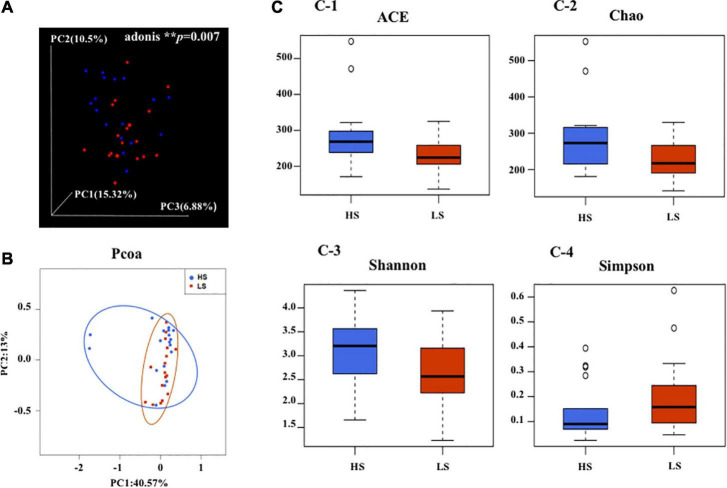
β-Diversity measures **(A,B)** and α-diversity measures **(C)** including **(C-1)** ACE, **(C-2)** Chao, **(C-3)** Shannon, and **(C-4)** Simpson of the fecal microbiota in HS and LS groups. HS, the high-status NCPB; LS, the low-status NCPB.

Overall, Adonis unweighted UniFrac dissimilarity metrics showed that the fecal microbial communities significantly differed with different statuses of NCPB (Adonis ^**^p = 0.007) (Figures 2A, B), suggesting dissimilar microbiota composition between the high-status and low-status groups. The level of α-diversity was slightly higher in the high-status group, but not statistically significant (Supplementary Table 3 and Figure 2C).

**FIGURE 3:**
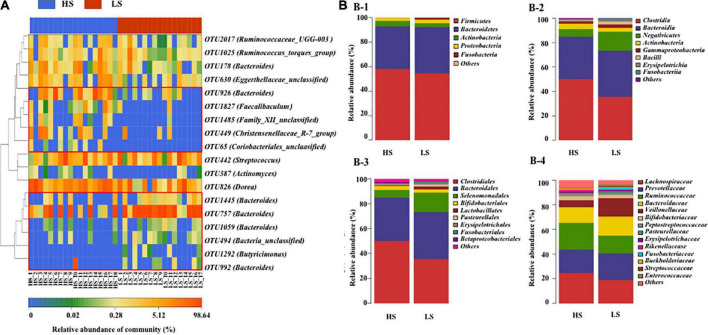
Heat map of relative abundance at the level of OTU **(A)** and composition **(B)** of the fecal microbiota at the level of **(B-1)** phylum, **(B-2)** class, **(B-3)** order, and **(B-4)** family in HS and LS groups. HS, the high-status NCPB; LS, the low-status NCPB.

In addition, the composition of the fecal microbiota was different in the high-status NCPB and the low-status groups. A total of 18 pivotal discriminatory OTUs were identified by using the Random Forest algorithm, including five OTUs (assigned to the genus of *Bacteroides*, *Faecalibaculum*, *Family_XIII_unclassified*, *Christensenellaceae_R-7_group*, and *Coriobacteriales_unclassified*) were overrepresented in the HS group, while six OTUs (assigned to the genus of *Bacteroides*, *Bacteria_unclassified*, and *Butyricimonas*) were overrepresented in the low-status NCPB group (Figure 3A). The taxonomic compositions of fecal microbiota in the two groups are shown in Figure 3B.

**FIGURE 4:**
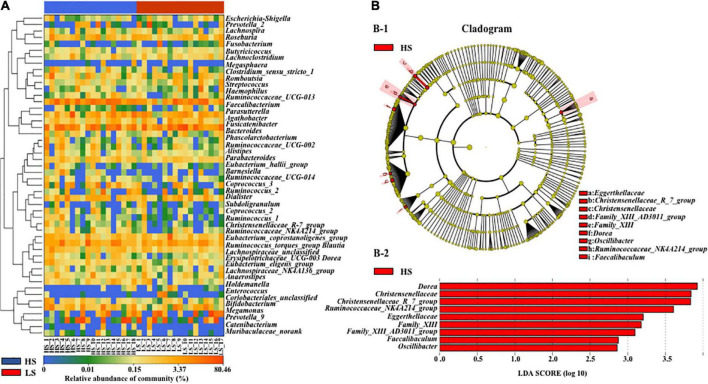
Heat map of 50 top genera in HS and LS groups **(A)** and difference of meaningful bacterial taxa **(B)** in HS and LS groups. **(B-1)** Taxonomic represents a statistical difference in groups. **(B-2)** Histogram of the LDA scores for differential abundant genera. HS, the high-status NCPB; LS, the low-status NCPB.

Further analysis revealed that several targets were significantly higher in the high-status group, including four families (*Family_XIII*, *Christensenellaceae*, *Peptococcaceae*, and *Eggerthellaceae*) and 12 genera (*Faecalibaculum*, *Family_XIII_unclassified*, *Ruminococcaceae_UCG-010*, *Ruminoco- ccaceae_unclassified*, *Eggerthellaceae_unclassified*, *Dorea*, *Chris- tensenellaceae_R7_group*, *Ruminococcaceae_NK4A214_group*, *Eub- acterium*, *Peptococcus*, *Family_XIII_AD3011_group*, and *Oscillibacter*) (Supplementary Table 4). The heat map of 50 top genera in HS and LS group was showed in Figure 4A.

Linear discriminant analysis effect size analysis [LDA scores (log10) >2] identified nine meaningful pregnancy taxa in the high-status NCPB group, inclusive of *Dorea*, *Christensenellaceae*, *Christensenellaceae_R_7_group*, *Ruminococcaceae_NK4A214_group*, *Eggerthellaceae*, *Family -_XIII*, *Family_XIII_AD3011_group*, *Faecalibaculum*, and *Oscillibacter* (Figure 4B).

### Shared bacteria associated with NCPB and depressive symptoms

As shown in Supplementary Table 5, young adults in the depressive symptom group also had more severe anxiety symptoms. No significant difference was found in gender, ethnicity, age and only-child status, and BMI between the depressive symptom group and normal controls.

**FIGURE 5:**
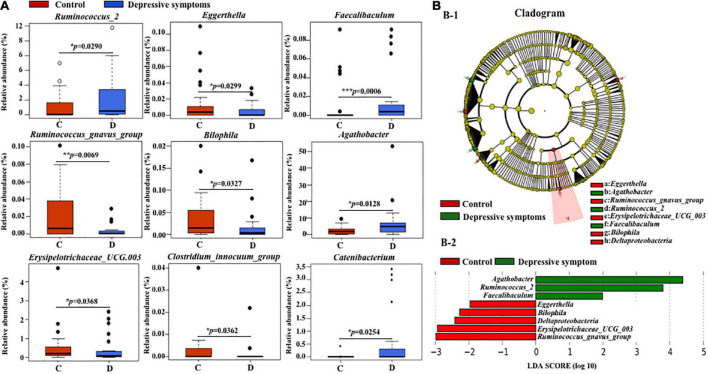
Boxplots of relative abundance of several genera with a significant difference in depression symptom and the control groups. **(A)** Difference of meaningful bacterial taxa in depression symptom and the control groups and **(B)** in depressive symptom and control groups. **(B-1)** Taxonomic represents a statistical difference in groups. **(B-2)** Histogram of the LDA scores for differentially abundant genera.

α-Diversity and β-diversity of the fecal microbiota were not significantly different, as shown in Supplementary Table 6 and Supplementary Figure 2A. Still, analyses revealed several taxon targets for the depressive symptom group, as shown in Figure 5 and Supplementary Figure 2B and Supplementary Table 7. LEfSe was also performed [LDA scores (log10) >1.5] and identified the meaningful taxa. *Agathobacter*, *Faecalibaculum* (LDA score was 1.9992248338), and *Ruminococcus_2* were significantly increased in the depressive symptom group, while *Bilophila*, *Eggerthella*, *Deltaproteobacteria*, *Erysipelotrichaceae_UCG_003*, and *Ruminococcus_gnavus_group* were decreased (Figure 5).

**FIGURE 6:**
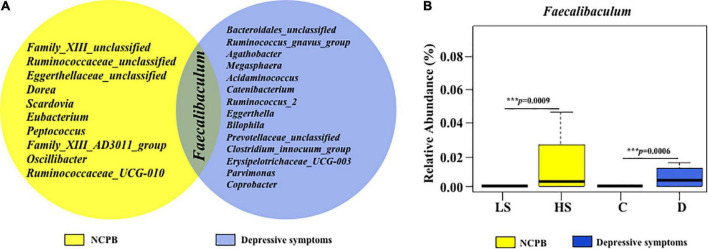
Analysis of commonly associated genera with NCPB and depressive symptom groups. **(A)** The common associated genera with NCPB and depressive symptom groups **(B)** as well as the results of *Faecalibaculum*. HS, the high-status NCPB; LS, the low-status NCPB; D, the depression symptom group; C, the control group.

The results of the fecal microbial structure indicated a significant fecal microbial imbalance in young male adults with a high level of NCPB or depressive symptoms. NCPB and depressive symptom groups shared similar bacteria (Figure 6A). *Faecalibaculum* was increased in both NCPB and the depressive symptom group, and a graphical representation is shown in Figure 6B.

### Predictive microbiota functional profiling and shared pathways associated with NCPB and depressive symptoms

**FIGURE 7:**
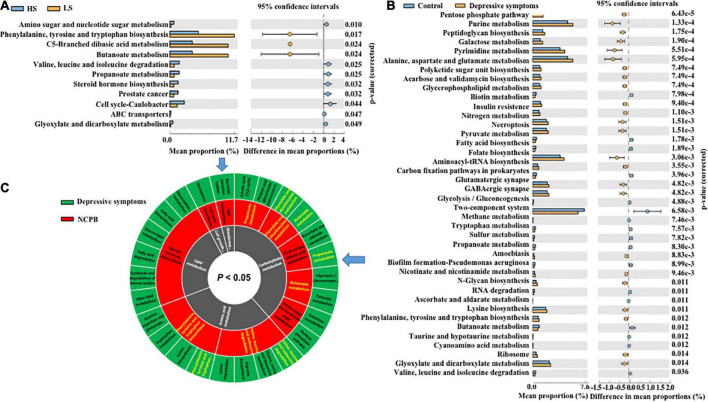
Analysis of functional pathways in NCPB groups and depressive symptom groups. **(A)** The high-status and low-status NCPB groups, **(B)** the depressive symptom and control groups, **(C)** and shared pathways associated with NCPB and depressive symptoms. HS, the high-status NCPB; LS, the low-status NCPB.

Functional profiling of microbial communities was predicted based on OTUs. A total of 11 pathways were found significantly different in the predictive microbiota functional profiling between the high-status and low-status NCPB groups. The predicted pathways mainly included compound metabolism (carbohydrate, amino acid metabolism, and lipid), membrane transport, cell growth, and death pathways. Detailed results are displayed in Figure 7A (*p* < 0.05).

Nevertheless, the predictive microbiota functional profiling was changed relative to depressive symptoms. In the functional pathway analyses, a total of 25 pathways were significantly enriched and 47 were depleted in the depressive symptom group. These pathways mainly included compound metabolism (e.g., carbohydrate, amino acid metabolism, nucleotide, and lipid), biosynthesis processes (e.g., lysine, arginine, fatty acid, phenylalanine, tyrosine, and tryptophan), degradation (e.g., lysine, fatty acid, and RNA), and signal transduction (e.g., two-component system), with detailed results partly presented in Figure 7B (*p* < 0.01) and whole in Supplementary Figure 3 (*p* < 0.05).

In addition, the results also showed that NCPB and depressive symptom groups shared similar pathways. Several common pathways related to both NCPB and depressive symptoms were also found including butanoate metabolism, glyoxylate and dicarboxylate metabolism, propanoate metabolism, phenylalanine, tyrosine and tryptophan biosynthesis, valine, leucine, and isoleucine degradation (Figure 7C).

## Discussion

### NCPB is positively correlated with depressive symptoms and anxiety symptoms

Depression is considered to be one of the most common mental disorders, and its pathogenesis is still unknown. In many cases, it is a common outcome of exposure to traumatic events, which influence information processing, modify attention or memory functions, and promote dysfunctional interpretations of current experiences (LeMoult and Gotlib, 2019; Qu et al., 2019; Vidańa et al., 2020). Referring to Beck’s model, cognitive distortions and negative shelf schema, often based on childhood experiences, bring about depression and co-occur with mood disorders (Sanchez et al., 2017; Beevers et al., 2019; Li et al., 2021; Noworyta et al., 2021). Apart from substantial research examining depressive cognitive content as a vulnerability factor (e.g., negative thoughts), a promising line of research highlights the role of NCPB in the development, maintenance, and relapse/recurrence of depressive symptoms or clinical depression (De Raedt and Koster, 2010; Dagmara et al., 2019; LeMoult et al., 2020).

In our studies, the relationship between NCPB, depressive symptoms, anxiety symptoms, and sleep quality among 735 Chinese young adults were discussed. In line with former studies, we certified that the scores on NCPBQ positively correlated with scores of depressive symptoms, anxiety symptoms, and sleep quality in Chinese young male adults. Further results in regression analyses showed that NCPB could positively predict the degree of depressive symptoms and anxiety symptoms and negatively predict sleep quality, covering the proportion of total variance of 16.37, 31.8, and 20.5%.

There is substantial evidence for general cognitive deficits in depression which is revealed in the literature (Joormann and Gotlib, 2010; Koster et al., 2011; Joormann and Vanderlind, 2014; Sanchez et al., 2017; Van den Bergh et al., 2018). Patients with cognitive deficits are typically characterized by impairments in attention, motor skills, working memory, and executive functions, which are prominent features of psychosis (LeMoult and Gotlib, 2019). Pathology-congruent interpretative biases are found in the prodromal phase; then, this presents an exciting new treatment possibility (Aronoriaga-Rodriguez and Fernandez-Real, 2019). Cognitive models have been proposed originally as etiological theories of depression (Mathews and MacLeod, 2005; Bomyea et al., 2017; Poole et al., 2017; Buckley et al., 2020).

### The composition of the microbiota is significantly associated with the level of NCPB

Nowadays, the microbiota–gut–brain axis has been proposed as a key regulator of stress responses, providing possibilities for the prevention and treatment of stress-related disorders (Gubert et al., 2020). The fecal microbiome seems to exert psychological effects affecting cognitive and emotional reactivity (Proctor et al., 2017; Vuong et al., 2017; Foster et al., 2021). Studies have found that disturbances in the homeostasis of the microbiota, such as a consequence of antibiotics, result in alterations at neural, hormonal, and immunological levels and are involved in the physiological stress response and behavior in both animals and humans, including patients with depression (Campos et al., 2016; Zheng et al., 2016; Sarkar et al., 2018). However, it is still unknown on the connection between the NCPB and the consequences of fecal microbial composition structure transform.

In our studies, we assessed the changes in the composition of the bacteria at different levels of NCPB. The significantly diverse fecal bacteria β-diversity and increase in the relative abundance of several microbiota, such as *Family_XIII*, *Christensenellaceae*, *Peptococcaceae*, and *Eggerthellaceae*, confirmed that the fecal bacteria comparison is variations of the alteration of the NCPB levels (Figure 2). We speculated that the degrees of our volunteers’ depressive symptoms might weaken the shift of microbial composition structure which has been verified in patients with MDD in previous studies (Ritchie et al., 2022). There were neither α-diversity nor β-diversity significant differences at the phylum level caused by aggravated depressive symptoms (Supplementary Figure 2A). Nevertheless, we found that some of the relative abundances of fecal microbiota, such as *Deltaproteobacteria*, had changed significantly, as shown in Figure 5. The cognition disorders involved in emotional disturbance, such as anxiety, have been associated with the imbalance of intestinal flora *via* the stress response in the clinical data (Ritchie et al., 2022). Several specific microbial families and genera have been associated with cognitive decline, anxiety behaviors, and affective disorders, such as *Bifidobacterium lactis CNCM I-2494*, *Lactobacillus bulgaricus*, *Streptococcus thermophilus*, and *Lactobacillus lactis*. The probiotic mixture containing these microbiota have been reported substantially to alter brain activity during the emotional reactivity test in healthy volunteers (Cryan and Dinan, 2012). To the best of our knowledge, how the gut microbiome might vary specifically from patients with depression with and without the NCPB specifier had not been explored previously.

### *Faecalibaculum* was the common bacterium associated with both NCPB and depressive symptoms

More interestingly, in our studies, among these flora community compositions converts, *Faecalibaculum*, which belongs to *Erysipelotrichaceae*, was the common bacterium associated with both NCPB and depressive symptoms at the genus level. It was not only higher in the high-state NCPB group but also in the depressive symptom group (Figure 6). It has been proven that *Faecalibaculum rodentium* transplanted from chronic social defeat stress (CSDS)-susceptible mice might induce the anhedonia-like behavior in the antibiotic cocktail (ABX)-treated WT and Ephx2KO mice, which did not show depressive behaviors in the exposure of CSDS. Ingestion of *Faecalibaculum* for 14 days induced anhedonia-like and depression behaviors of ABX-treated Ephx2KO mice, accompanied by the increased expression of proinflammatory factors, such as interleukin-6 in the blood and reduction of synaptic proteins expression in the prefrontal cortex (Wang et al., 2021). The combination of probiotics and prebiotics contains *Lactobacillus* with *Faecalibaculum*, *Blautia*, or *Bifidobacterium* spp. was proven to normalize the gut microbiome diversity and improve depressive-like behavior of the chronic stress-induced depression and anxiety in mice model (Westfall et al., 2021). Furthermore, growing evidence indicates that *Faecalibaculum* enriched “Western diet” or high-fat diet (HFD) fed mice, especially positively correlated with serum proinflammatory cytokines such as TNF-α, IL-6, and LBP in HFD-fed mice, and closely related to the metabolism disorders involved in energy production or adiposity (Skonieczna-Żydecka et al., 2018; Ma et al., 2019; Wei et al., 2020). *Faecalibaculum* was considered a gut antigen causing the abnormal function of the microbiota–gut–brain axis (Oleskin and Shenderov, 2016; Dalile et al., 2019). A high sugar diet and innate lymphoid cell 3 (ILC3) promoted that the outgrowth of *F. rodentium* could displace Th17-inducing microbiota and posing risk for metabolic syndrome in mice (Kawano et al., 2022). Activity ileal ILC3 might regulate the ileal Treg/T helper 17 cells ratio and impact the production of hippocampal and prefrontal cortex chemotactic in the stress-induced behavioral deficits mice model, which could be relieved by the combination of probiotics and prebiotics and promoted behavioral resilience to the chronic and recurrent stress by normalizing gut microbiota populations (Westfall et al., 2021). These studies have revealed a connection between stress-induced depression and anxiety-like behavioral impairments depending on the microbiota–gut–brain axis and immune regulation system, and most of them were based on animal research rather than clinical studies.

### Shared metabolic pathways associated with NCPB and depressive symptoms were mainly involved with SCFAs

We continuously analyzed the functional profiling of microbiota communities predicted based on OTUs. It is revealed that 11 functional pathways were significantly different and mainly focused on compound metabolisms, such as carbohydrate, amino acid metabolism and lipid, membrane transport, cell growth, and death pathways (Figure 7A). It has been proven that the production of several metabolic responses impacts a variety of life functions, including neurological functions (Kennedy et al., 2017; Martin et al., 2020).

The pathways, including the phenylalanine, tyrosine and tryptophan biosynthesis, butanoate metabolism, propanoate metabolism, glyoxylate and dicarboxylate metabolism, were significantly altered between the high-status and low-status NCPB in our studies. And they were reported to be involved into the cognitive dysfunction in rodents and patients with depression. In which five pathways, such as butanoate metabolism, glyoxylate, and dicarboxylate metabolism, propanoate metabolism, phenylalanine, tyrosine, and tryptophan biosynthesis, valine, leucine, and isoleucine degradation, were invovled with environment responses such as two-component system, and the neurotrophy relevantly pathways such as folate biosynthesis and biosynthesis of amino acids. The production of these pathways, especially the SCFAs, such as butanoate, phenylalanine, tyrosine, and tryptophan, has been proven to modulate multiple biological processes including neurological function. Produced by the most endogenous microbiota, SCFAs readily pass through the mucosa layer and cell membranes and exert toxic effects on mammal cells at high concentrations, particularly affecting the functions of the nervous system by serving as nutrients, metabolites, or regulators involved in the operation of various kinds of nervous cells (Valles-Colomer et al., 2017).

A clear association has been found between lower levels of SCFAs and decreased representation of obligate anaerobes such as the *Faecalibaculum*, *Lachnospiraceae*, and *Ruminococcaceae* in human fecal microbiota (D’Amato et al., 2020). The fecal microbiota transplant contained those microbiotas including *Faecalibaculum* and *Lachnospiraceae* from aged donor mice into young adult recipients altered the abundance of bacteria associated with SCFAs production and impacted the cognitive function (D’Amato et al., 2020). Several studies have revealed the potential ability of *Erysipelotrichaceae*, *Bifidobacterium*, *Faecalibaculum*, *Bacteroides*, and *Romboutsia* to produce SCFAs in mice (Smith et al., 2013; Dalile et al., 2019). A correlation between SCFA levels and the abundance of *Faecalibaculum*, *Romboutsia*, *Bacteroides*, and *Turicibacter* were found in rats. *Faecalibaculum* was the most strongly positively correlated with the levels of SCFA levels, which are well known as the endogenous ligands of PPARγ, and crucially together with increasing PPARγ expression, promoting the PPARγ/MAPK/NF-κB signaling pathway connected with the metabolism and immune system (Zhang et al., 2022).

In addition, SCFAs, especially butyrate production, provide energy substrates for colonocytes, mitigate inflammation, and regulate satiety for their host. Their deficiency or redundancy not only leads to metabolic diseases but is also involved in depression and other mood disorders (Oleskin and Shenderov, 2016). Michels et al. (2017) and Skonieczna-Żydecka et al. (2018) demonstrated that emotional problems in Belgian children and Polish depressive women. were associated with a significantly higher concentration of butyrate, isobutyrate, valerate, and isovalerate. Hence, the results of fecal microbial structure and function alteration indicated a significant fecal microbial imbalance in Chinese young male adults with high levels of NCPB with depressive symptoms. It may be a hint that explains the mechanism of higher levels of NCPB involved with severe depressive symptoms.

## Conclusion

This is the first study on the connection between microbiota and NCPB, exploring the co-exist microbiota of NCPB and depressive symptoms by sequencing and analyzing the fecal microbiota of 735 Chinese young adults who have to take a balanced diet for 1 month and restricted of snacks. The NCPB status of these young adults was positively correlated with the degree of depressive symptoms as well as anxiety symptoms and sleep quality (*p* < 0.01). We, fortunately, found several target bacteria taxa, including *Dorea*, *Christensenellaceae*, *Christensenellaceae_R_7_group*, *Ruminococcaceae_NK4A214_group*, *Eggerthellaceae*, *Family-XIII*, *Family_XIII_AD3011_group*, *Faecalibaculum*, and *Oscillibacter* that were significantly enriched in the high-status NCPB group than in the low-status group. These bacteria taxa were mainly involved in pathways including SCFA metabolism. Furthermore, *Faecalibaculum*, which was proven involved with the depressive-like phenotypes in laboratory animals, was also found significantly increased in the depressive symptom group compared with the control group. Five pathways, such as butanoate metabolism, glyoxylate, and dicarboxylate metabolism, turned out to be significant altered in both the high-status NCPB group and the depressive symptoms group. The results hint at the common bacteria taxa and pathways involved with either NCPB or depressive symptoms and provided some clues for the crosstalk of NCPB and depressive symptoms *via* the microbiota–brain–gut axis.

## Limitations

There was an exploratory study that needs to be replicated across larger samples and compared with a healthy control group.

## Data availability statement

The data presented in this study are deposited in the BIG submission, the accession numbers are CRA009867 and CRA009825. Available from https://ngdc.cncb.ac.cn/gsa/s/m0k5fYEp and https://ngdc.cncb.ac.cn/gsa/s/OD562MZb.

## Ethics statement

The studies involving human participants were reviewed and approved by the study of the mechanism of overgeneralized autobiographical memory in three stages of suicide in depression. The studies described in our manuscript is part of the Ethics Committee. The patients/participants provided their written informed consent to participate in this study.

## Author contributions

Z-ZF guided the studies and critically revised the manuscript. XH conceived and designed the experiments. S-WX, H-MX, T-YL, and XH collected and analyzed the data. XH, H-MX, and XZ wrote the manuscript. All authors contributed to the article and approved the submitted version.

## Footnotes

1
http://qiime.org/

2
http://www.genome.jp/kegg/pathway.html
