# Supplementary material for: Microbiota alteration of Chinese young male adults with high-status negative cognitive processing bias
Source: Front Microbiol. 2023 Mar 1;14:989162. doi: 10.3389/fmicb.2023.989162 (PMC10015002; doi:10.3389/fmicb.2023.989162)
Supplement: Supplementary file 1 [file Data_Sheet_1.docx]

***Supplementary Material***

**Supplementary Table 1**

**Table 1** The socio-demographics characteristics of 735 participants and difference analysis of study variables in categorical items (M±SD)

| Variable | Frequency *n*=735 | Percent(%) | 95%CI (%) | NCPB | Depressive symptoms | Anxious symptoms | Sleep quality |
| --- | --- | --- | --- | --- | --- | --- | --- |
| Gender |  |  |  |  |  |  |  |
| Male | 735 | 100.00% | 100.00-100.00 | 37.11±10.74 | 0.44±0.10 | 38.68±8.41 | 5.05±2.15 |
| Only-child status |  |  |  |  |  |  |  |
| Yes | 196 | 26.67% | 23.54-29.66 | 37.44±10.83 | 0.44±0.10 | 39.02±7.93 | 5.65±3.23 |
| No | 539 | 73.33% | 70.34-76.50 | 36.99±10.71 | 0.44±0.11 | 38.55±8.58 | 4.84±2.87 |
| *t* | — | — | — | 0.51 | -0.62 | 0.66 | 3.07** |
| Ethnicity |  |  |  |  |  |  |  |
| Han | 730 | 99.32% | 98.64-99.86 | 37.09±10.75 | 0.44±0.10 | 38.61±8.38 | 5.04±2.99 |
| Minority | 5 | 0.68% | 0.14-1.36 | 40.20±8.70 | 0.55±0.14 | 48.20±8.96 | 7.80±1.92 |
| *t* | — | — | — | -0.65 | -2.35* | -2.55* | -2.07* |

**p*<0.05, ** *p*<0.01

**Supplementary Table 2**

**Table 2** Characteristics of subjects

| Variable | HS (*n*=18) | LS (*n*=17) | *p* |
| --- | --- | --- | --- |
| Gender | Male | Male | — |
| Ethnicity | Han | Han | — |
| Age, M±SD | 21.16±1.20 | 22.00±1.46 | 0.06 |
| Only-child status, *n*(%) | 27.8 | 17.6 | 0.48 |
| BMI | 21.17±1.68 | 21.61±1.93 | 0.48 |
| Depressive symptoms, M±SD | 0.55±0.11 | 0.44±0.13 | 0.03 |
| NCPB, M±SD | 59.56±11.04 | 21.94±1.09 | <0.01 |
| Anxious symptoms, M±SD | 50.00±11.69 | 36.00±6.73 | <0.01 |
| Sleep quality, M±SD | 7.36±3.35 | 2.49±1.95 | <0.01 |

HS:The high-status of NCPB; LS:The low-status of NCPB

**Supplementary Table 3**

**Table 3** Alpha diversity of microbiota in HS and LS groups

| Alpha diversity | HS | LS | *p* |
| --- | --- | --- | --- |
| ace | 282.94±92.17 | 235.04±54.15 | 0.11 |
| chao | 286.71±94.27 | 232.70±53.92 | 0.10 |
| shannon | 3.08±0.69 | 2.64±0.71 | 0.06 |
| simpson | 0.14±0.11 | 0.20±0.15 | 0.09 |

HS:The high-status of NCPB; LS:The low-status of NCPB.

**Supplementary Table 4**

**Table 4** Difference in microbiota at family and genus level in HS and LS groups

| Levels | HS | LS | *p* | Enrichment |
| --- | --- | --- | --- | --- |
| Family level |  |  |  |  |
| *Family_XIII* | 6.67E-4±9.23E-4 | 1.44E-4±2.25E-4 | <0.01 | HS |
| *Christensenellaceae* | 4.17E-3±1.04E-2 | 2.42E-3±8.02E-3 | 0.03 | HS |
| *Peptococcaceae* | 5.60E-5±1.15E-4 | 1.85E-6±7.63E-6 | 0.04 | HS |
| *Eggerthellaceae* | 1.46E-3±1.85E-3 | 5.91E-4±5.70E-4 | <0.05 | HS |
| Genus level |  |  |  |  |
| *Faecalibaculum* | 1.99E-4±3.18E-4 | 3.15E-5±1.15E-4 | <0.01 | HS |
| *Family_XIII_unclassified* | 2.62E-4±3.08E-4 | 2.59E-5±6.97E-5 | <0.01 | HS |
| *Ruminococcaceae_UCG-010* | 7.15E-4±2.04E-3 | 1.85E-6±7.63E-6 | <0.01 | HS |
| *Ruminococcaceae_unclassified* | 2.32E-3±3.18E-3 | 1.08E-3±2.68E-3 | 0.02 | HS |
| *Eggerthellaceae_unclassified* | 1.19E-3±1.79E-3 | 3.59E-4±5.23E-4 | 0.02 | HS |
| *Dorea* | 8.09E-3±9.21E-3 | 4.31E-3±4.94E-3 | 0.03 | HS |
| *Scardovia* | 0 | 7.41E-6±1.38E-5 | 0.03 | LS |
| *Christensenellaceae_R-7_group* | 4.04E-3±1.01E-2 | 2.36E-3±7.75E-3 | 0.03 | HS |
| *Ruminococcaceae_NK4A214_group* | 2.92E-3±6.19E-3 | 8.09E-4±2.29E-3 | 0.04 | HS |
| *Eubacterium* | 1.22E-5±2.67E-5 | 0 | 0.04 | HS |
| *Peptococcus* | 3.67E-5±9.97E-5 | 0 | 0.04 | HS |
| *Family_XIII_AD3011_group* | 2.26E-4±3.78E-4 | 6.66E-5±1.39E-4 | 0.04 | HS |
| *Oscillibacter* | 2.85E-4±5.92E-4 | 1.22E-4±3.08E-4 | <0.05 | HS |

HS:The high-status of NCPB; LS:The low-status of NCPB.

**Supplementary Table 5**

**Table 5** Characteristics of subjects

| Variable | Depressive symptom group (*n*=27) | Control group (*n*=33) | *p* |
| --- | --- | --- | --- |
| Gender | Male | Male | — |
| Ethnicity | Han | Han | — |
| Age, M±SD | 21.37±1.76 | 22.18±1.63 | 0.06 |
| Only-child status, n(%) | 22.2 | 18.2 | 0.62 |
| BMI | 21.36±1.91 | 21.67±2.07 | 0.56 |
| Depressive symptoms, M±SD | 0.61±0.08 | 0.41±0.06 | <0.01 |
| NCPB, M±SD | 44.89±19.79 | 33.70±11.02 | 0.01 |
| Anxious symptoms, M±SD | 48.11±9.55 | 34.89±5.52 | <0.01 |
| Sleep quality, M±SD | 5.58±3.69 | 4.12±2.79 | 0.17 |

**Supplementary Table 6**

**Table 6** Alpha diversity of the microbiota in depressive symptoms and control groups

| Alpha diversity | Control group (*n*=33) | Depressive symptoms group (*n*=27) | *p* |
| --- | --- | --- | --- |
| ace | 361.28±102.77 | 342.56±75.03 | 0.53 |
| chao | 365.60±104.43 | 343.98±84.36 | 0.45 |
| shannon | 3.14±0.67 | 3.01±0.65 | 0.44 |
| simpson | 0.13±0.14 | 0.14±0.09 | 0.39 |

**Supplementary Table 7**

**Table 7** Difference in the microbiota at genus level in depressive symptoms and control groups

| Genus | C | D | *p* | Enrichment |
| --- | --- | --- | --- | --- |
| *Faecalibaculum* | 7.36E-5±2.08E-4 | 1.56E-4±2.79E-4 | <0.01 | D |
| *Bacteroidales_unclassified* | 8.61E-4±1.35E-3 | 1.88E-4±3.01E-4 | <0.01 | C |
| *Ruminococcus_gnavus_group* | 2.20E-3±2.97E-3 | 3.96E-4±6.85E-4 | <0.01 | C |
| *Agathobacter* | 2.45E-2±2.49E-2 | 7.05E-2±0.11 | 0.01 | D |
| *Megasphaera* | 2.11E-4±1.17E-3 | 3.73E-3±9.35E-3 | 0.02 | D |
| *Acidaminococcus* | 0 | 1.46E-4±4.91E-4 | 0.02 | D |
| *Catenibacterium* | 1.30E-4±7.32E-4 | 5.00E-3±1.07E-2 | 0.03 | D |
| *Ruminococcus_2* | 9.94E-3±1.64E-2 | 2.23E-2±3.06E-2 | 0.03 | D |
| *Eggerthella* | 1.36E-4±2.43E-4 | 5.11E-5±8.96E-5 | 0.03 | C |
| *Bilophila* | 3.94E-4±5.22E-4 | 1.83E-4±3.69E-4 | 0.03 | C |
| *Prevotellaceae_unclassified* | 8.68E-5±2.59E-4 | 2.54E-2±0.12 | 0.03 | D |
| *Clostridium_innocuum_group* | 2.97E-5±7.11E-5 | 1.21E-5±4.27E-5 | 0.04 | C |
| *Erysipelotrichaceae_UCG-003* | 5.05E-3±8.55E-3 | 3.98E-3±6.76E-3 | 0.04 | C |
| *Parvimonas* | 5.28E-5±2.29E-4 | 1.34E-6±6.98E-6 | <0.05 | C |
| *Coprobacter* | 1.17E-3±5.11E-3 | 2.82E-5±8.73E-5 | <0.05 | C |

D:The depressive symptoms group; C: The control group.

**Supplementary Figure 1**


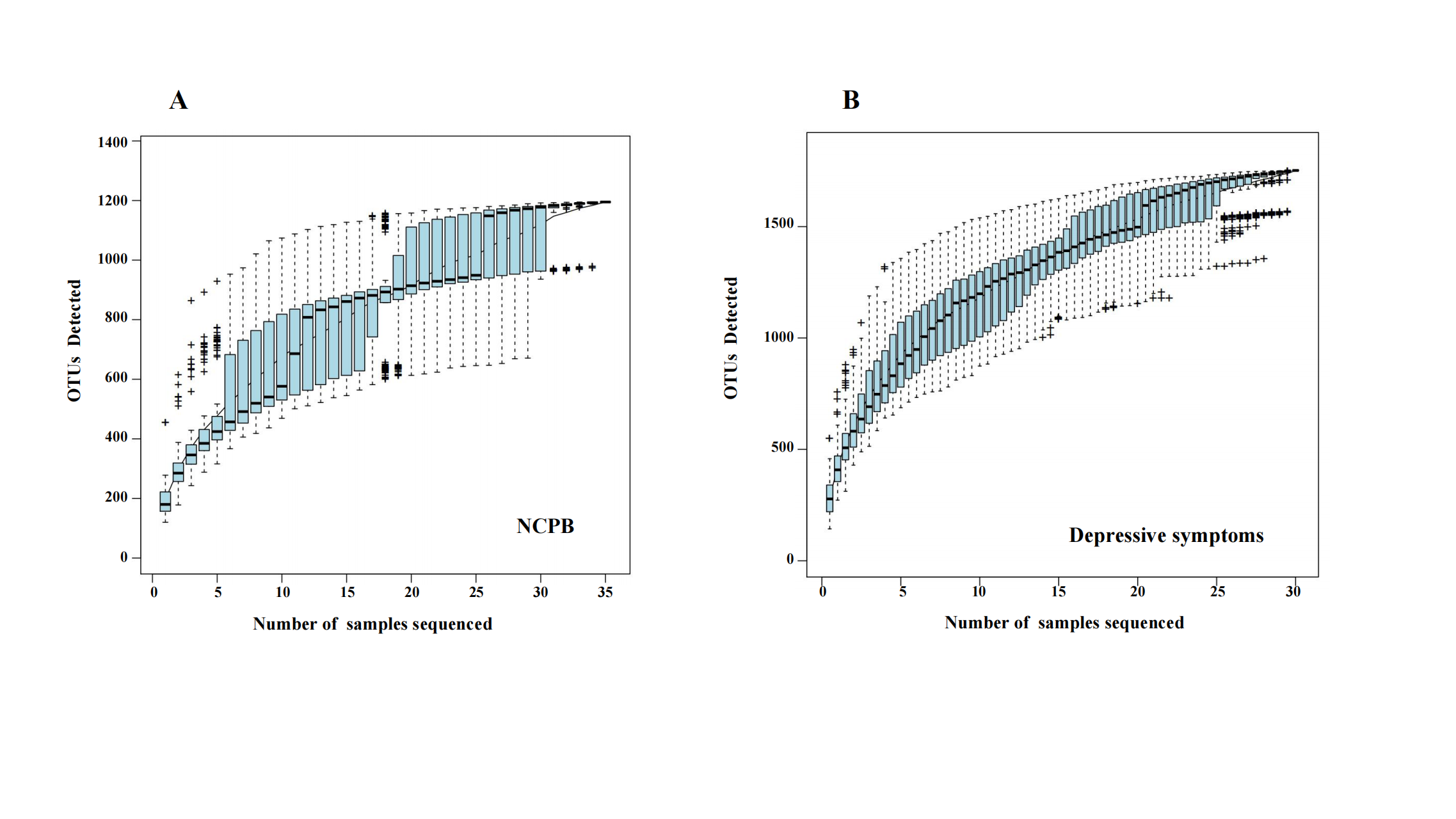


**Supplementary Figure 1** Analysis for the sample size of flora analysis and the data collection volume in NCPB and depressive symptoms groups.

**Supplementary Figure 2**


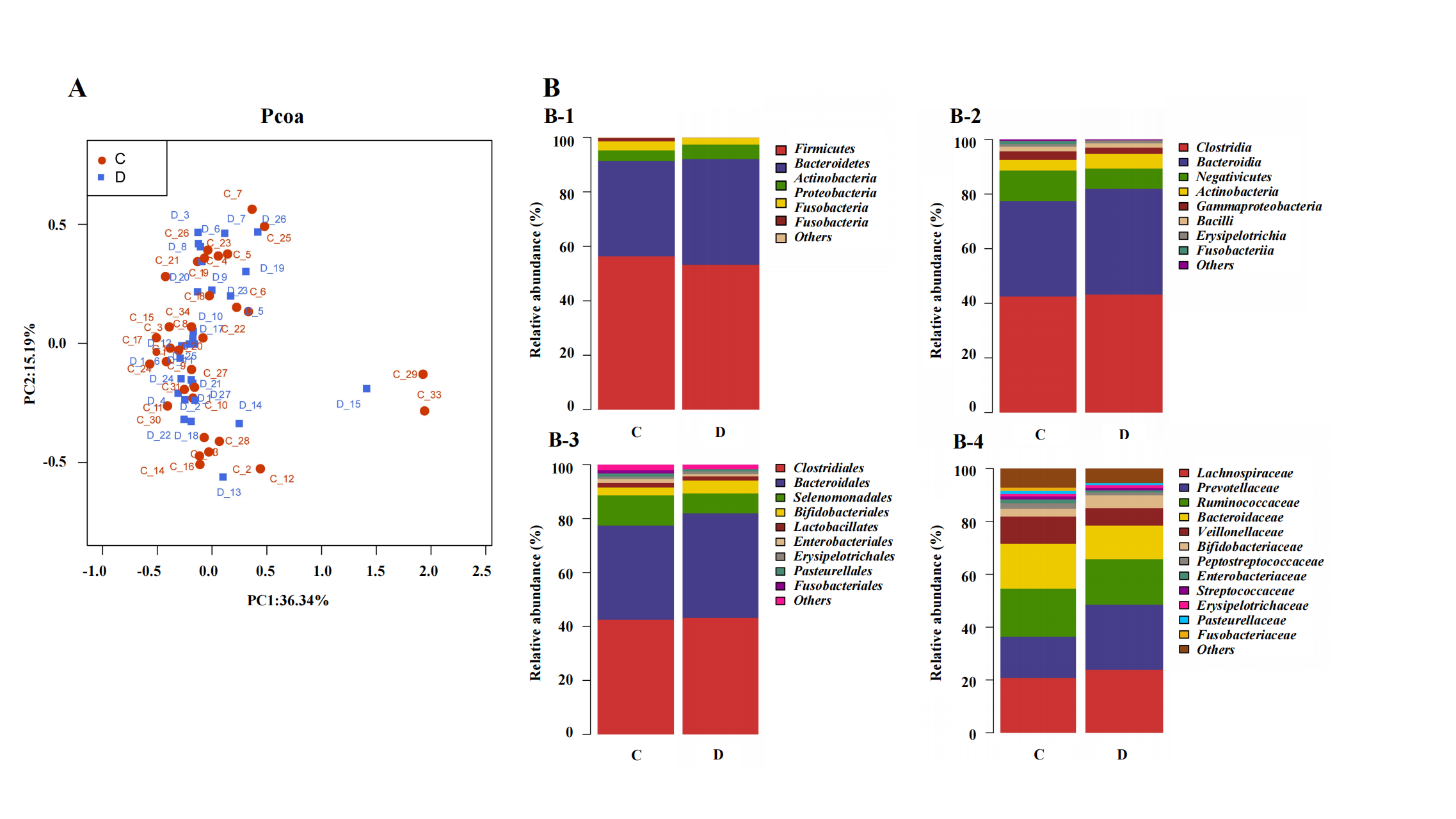


**Supplementary Figure 2** Beta diversity measures **(A)** and composition of the gut microbiota **(B)** in depressive symptoms and control groups at the level of **(B-1)** phylum, **(B-2)** class, **(B-3)** order and **(B-4)** family.

D:The depressive symptoms group; C: The control group.

**Supplementary Figure 3**


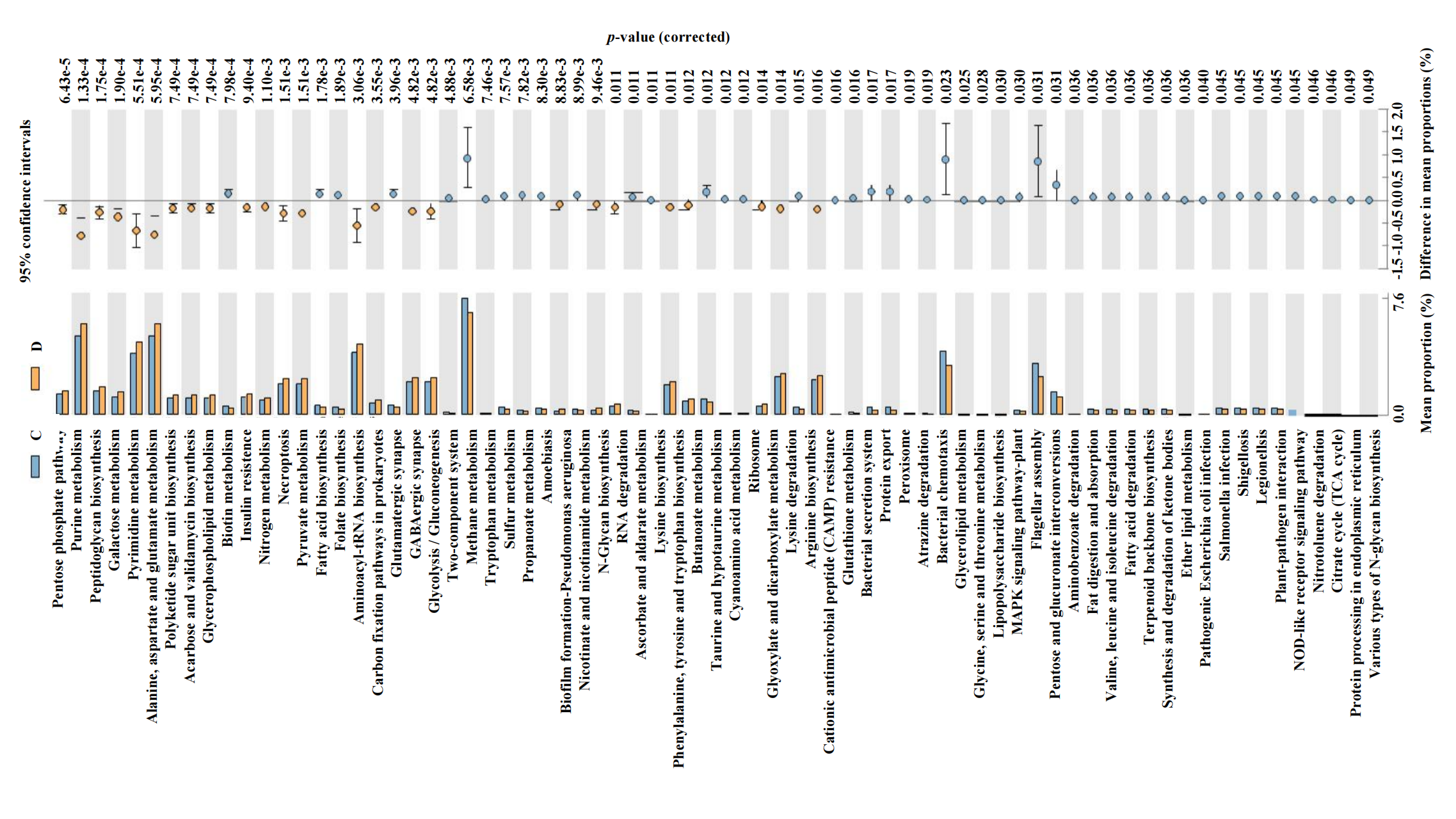


**Supplementary Figure 3** Analysis for functional pathways in depressive symptoms and control groups (*p*<0.05).

D:The depressive symptoms group; C: The control group.
